# Sexual dysfunction and associated factors among patients with epilepsy at Amanuel Mental Specialty Hospital, Addis Ababa – Ethiopia

**DOI:** 10.1186/s12883-019-1432-1

**Published:** 2019-10-27

**Authors:** Alem K. Ejigu, Kelemwa H. Zewlde, Niguse Y. Muluneh, Zehara R. Seraj, Mahlet W. GebreLibanos, Yodit H. Bezabih

**Affiliations:** 1 Addis Ababa, Ethiopia; 2 UOG, Gondar, Ethiopia

**Keywords:** Sexual dysfunction, Epilepsy, Ethiopia, Amanuel hospital

## Abstract

**Background:**

Many patients with chronic illness have sexual dysfunction that may be related to the condition itself, drug side effects, emotional sequel, or a combination of those factors. Patients with epilepsy are no exception. Men and women with epilepsy are frequently complaining sexual dysfunction and they appear to have a higher incidence of sexual dysfunction than peoples with other chronic neurologic illness. These problems can have a substantial impact on their sexuality thus; it needs careful study and evaluation.

**Methods:**

Hospital based cross sectional study was conducted from January to July, 2016 among Patient with Epilepsy at Amanuel Mental Specialty Hospital. Interviewer administered Changes in Sexual Functioning Questionnaire (CSFQ-14) was used in order to assess the sexual problems. Finally, the data was analyzed by using Statistical Package for Social Science (SPSS) V-20. Descriptive statistics and logistic regression were used to describe the variables. Levels of significance of association determined at < 0.05.

**Results:**

A total of 694 respondents participated, with response rate 99.14%. Among them 576 completed all items. The result showed that 363 subjects (63.9, 95%CI = 59.5–67.7) had global sexual dysfunction. Furthermore, the rate of sexual dysfunction was reported as 55.6% (95%CI = 49.1–62.6) and 67.4% (95%CI = 62.8–72.1) in female and male participants, respectively. Among domains of sexual dysfunction; sexual arousal problem (97.8% (95%CI = 95.8–98.3)) and sexual pain problem (11.3% (95%CI = 8.8–13.9)) were the most and the least prevalent sexual dysfunctions respectively. Concerning associated factors; age grouped > 51, depression, being out of relationship or not satisfied with non-sexual aspect of relationship, being jobless and khat use has positive association with sexual dysfunction. By the other side alcohol use, level of education & age groups 18–21 years associated negatively.

**Conclusions:**

The prevalence of sexual dysfunction among patients with epilepsy is very high; its prevalence is more among males than females. Sexual arousal problem and sexual pain problem were the most and the least prevalent sexual dysfunctions respectively.

**Electronic supplementary material:**

The online version of this article (10.1186/s12883-019-1432-1) contains supplementary material, which is available to authorized users.

## Background

The quality of human life is a summation of many factors. Male and female sexuality, relationships and successful reproduction are paramount in the accomplishment of a meaningful life for most individuals [1–3]. Sexual function is the physiological capacity to experience desire, arousal, and orgasm. Sexual dysfunctions are a heterogeneous group of disorders that are typically characterized by clinically significant disturbances in a people’s ability to respond sexually or to experience sexual pleasure [4, 5].

Sexual dysfunction as a disorder is complex and often multi factorial, with medical, psychological, and life circumstances all playing important roles [6–9]. Epilepsy (a chronic neurologic disorder the hallmark of which is recurrent, unprovoked seizures) is one of neurological disorder which can cause sexual dysfunction. Sexual disorders (both hypo-sexuality and sexual dysfunction) are common in patient with epilepsy, occurring in up to two thirds of patient with epilepsy (PWE) [10]. Men and women with epilepsy frequently complain, if asked, of sexual dysfunction and appear to have a higher incidence of sexual dysfunction than peoples with other chronic neurologic illness.

The aim of this cross-sectional study was to assess the magnitude of sexual dysfunction and factors associated with it among PWE at Amanuel Mental Specialty Hospital, Addis Ababa, Ethiopia. The findings of this study could be useful evidence for designing proper intervention and possible managements for professionals and responsible organs.

## Methods

### Study design and study period

Institutional based cross sectional study was conducted from May 15 to June 30, 2016.

### Study area

An institution based study was conducted at AMSH, AA - Ethiopia. The hospital has different teams. Neuro Epileptic Case Team (NEP) is one of health service team in the hospital. There are about 34,210 PWE who have regular follow up in a year at hospital’s NEP clinics. Among them 11,634 (34%) are females [11]. Averages of 1900 cases are seen monthly (AMSH, Central statistic bureau). The area is selected because of high patient flow which has good opportunities to trace patients who can be representative for the study population.

### Population

#### Source population

Our source populations were all PWE who present for follow up visit at AMSH, NEP clinics.

#### Study population

Our study populations were PWE in the age group 18 and above who were presented for follow-up visit at AMSH NEP clinics in the study period.

### Eligibility criteria

PWE age 18 and above, who had sexual experience for six or above months were included in this study.

PWE who is on ectal or post-ectal phase were not included in this study.

### Sample size & sampling procedure

#### Sample size

The minimum number of sample required for this study determined by using power population proportion formula considering the following assumptions;

95% confidence level, 50% sexual dysfunction and 5% margin of error

1 $$ n=\frac{{\left(\frac{Z\alpha}{2}\right)}^2p\left(1-P\right)}{d^2} $$

$$ n=\frac{(1.96)^20.5\left(1-0.5\right)}{(0.05)^2}=384 $$

In Ethiopian culture, openly discussing on issues related with sexuality is taboo and not common. By considering such conditions and other studies non respondent rate this study was used a maximum non respondent rate; 15%. Thus; the total sample size calculated with single population proportion to study prevalence of sexual dysfunction among PWE.

2 $$ 384+\left(384\ast 15\%(384)\right)=442. $$

To address associated factors, final sample size was calculated by using power population proportion as:

3 $$ 608+\left(608\ast 15\%\left(\ 608\right)\right)=608+91.2=699.2\approx 700 $$

#### Sampling procedure

Our study participants were select from AMSH, NEP OPDs through stratified systematic random sampling technique. Sample size was allocated proportionally for each sex categories (66% for male and 34% for female participants). Sampling interval was determined by dividing total study population; 2850 PWEs [11]; who had follow up during six weeks data collection period by total sample size (700). The sampling fraction was: 2850/700 = 4.07. Approximately five was taken not to miss respondents in the sampling fraction above four (0.07). The first respondent was selected by lottery method from the first five study population for each strata, and the next respondent was chosen at regular intervals of every 5th cases in each sex categories.

### Instruments used for data collection and data collection procedures

#### Instruments used for data collection

In this study, to assess Socio-demographic information, clinical data, quality of sexual functioning and factors associated with sexual dysfunction; We mainly used Couples Satisfaction Index (CSI) scales to assess participant’s satisfaction on their relationship, Daily Stress Full Life Events Measurement Scale (DSLEMS) to assess presence of Stressful Life Events and to obtain socio-demographic and clinical data a detailed self-designed semi structured questionnaire was administered to study participant.

Sexual response was measured by using Changes in Sexual Functioning Questionnaires (CSFQ-14-) which have separate forms for female (CSFQ - Female Clinical Version) and male (CSFQ - Male Clinical Version) data. CSFQ has 14 items on a single sheet and it is used to assess the existence of sexual problems in study participants. All the 14 items should be answered on a five point (Likert type) scale to assess global sexual dysfunction. Eleven of the items from “Never”, through “Rarely”, “Sometimes”, and “Often”, to “Every day”; the rest three of the items which has reverse scored from No”, through “Little”, “Some”, and “much”, to “Great”. In addition other CSFQ domains; Sexual Desire, Pleasure, arousal/excitement and Orgasm/Completion can be obtain and represent as a profile. The CSFQ-14- has chosen because it is standard, have a brief, valid, reliable, relatively unobstructive, available and gender-specific questionnaire to monitor sexual functioning. Also it has Cronbach’s α 0.91 and 0.93 for the male and female scales, respectively [12].

The English version of instruments was translated to Amharic language and retranslate to English by accepted psychiatric professionals. For participants in different languages, translators were assisting data collectors.

#### Data collection procedures

Data collectors got brief orientation on the data collection procedure and data collection protocol before they engage into actual data collection activities. Three health officers and three BSC nurses (four males and two females) were recruited to collect data. Amharic Version of structured and semi structured interviewer administered questionnaire was used. It has six parts: The 1st part contains socio-demographic characteristics of participants; the 2nd part contains “Changes in Sexual Functioning Questionnaires (CSFQ)”; the ^3rd^ and the 4th parts were containing Relationship Assessment Scale (RAS) and Self-esteem scale respectively, Daily Stressful Life Events Measurement Scale (DSLEMS) was the 5th tool used to assess Stressful Life Event/s and the last part contains clinical data of participants. This tools were administered to 700 eligible clients at AMSH; NEP clinics during the study period. Privacy of participants was given special consideration before the beginning of data collection. To make the communication easy female data collectors were expected to contact female subjects and the same procedure were performing for male subjects. Two separate OPDs (NEP OPD-3rd for males and MCH OPD for females) were used to obtain data from each sex categories.

### Definition of variables

Sexual Dysfunction was dependent variable and independent variables were Psychosocial factors (quality of relationship, stressful life event/s, self-esteem), Demographic factors (age, sex, education, occupation, relationship status), Clinical factors (comorbid conditions, medication for comorbidities, obesity), Illness related factors (type of AEDs, seizure experience (uncontrolled/ controlled), age onset of epilepsy, medication regimen, type of epilepsy], Behavioral factor (alcohol use, khat use or/and cigarette use).

**Sexual dysfunction**: it is explained by total scores below the cutoff points from all 14 CSFQ elements; below 41 and 47 for female and male participants respectively [12].

**Sexual dissatisfaction**: it is explained by scoring less than 5 from CSFQ-14- (item one).

**Sexual desire problem**: it is explained by scoring less than 16 for females and less than 20 for males from the sum of CSFQ-14- (items 2 through 6).

**Arousal/excitement dysfunction**: it is explained by scoring less than 13 for females and less than 14 for males from the sum of CSFQ-14- (items 7 through 9).

**Anorgasmia**: it is explained by scoring less than 12 for females and less than 14 for males from the sum of CSFQ-14- (items 11 through 13).

**Dyspareunia**: it is explained by scoring less than 5 for both sex categories from the CSFQ − 14- (item teen).

**Quality of relationship**: it is explained by score above 15 from the summation of Relationship Assessment Scale called the more satisfied with his/her relationship [8, 13] .

**Poor self-esteem**: is defined as score below 15 on Rosenberg self-esteem scale.

**Khat**: is a flowering plant native to the Horn of Africa and the Arabian Peninsula. It contains the alkaloid cathinone, a stimulant, which is said to cause excitement, loss of appetite and euphoria [14].

**Comorbid illness**: is defined as presence of additional illness among PWE; which includes previously confirmed psychosis, depression, hypertension, diabetic mellitus, and asthma.

**Stressful Life Event**: experiencing one or more from listed ten items within the past six months from time of data collection.

**Poly therapy**: taking two or more than two types of AEDs.

**Underweight**: BMI less than 18.5 Kg/m^2^.

**Normal weight**: BMI in the range 18.5 to 24.99 Kg/m^2^.

**Overweight**: BMI in the rages of 25 to 29.99 Kg/m^2^.

## Data processing and analysis

Data was code and entered to Epi-Info version 7 for cleaning, storing and recording. It was imported to computer software Statistical Package for Social Sciences (SPSS) version 20. Descriptive statistical procedures was utilize to estimate the prevalence of sexual dysfunction and to describe other variables. Bivariate and multivariable logistic regression analyses were conducted to identify associated factors of sexual dysfunction among PWE. The strength of the associated factors was presented by odds ratio with 95% CI. The independent variables that fulfill *p*-value less than 0.2 were tolerated and exported to multivariable logistic regression. *P*-value less than 0.05 were considered as statistically significance.

The data was mainly analyzed as global sexual dysfunction and factors associated with sexual dysfunction. It also reported separately based on sex categories and in each domains of sexual dysfunction in each sex categories.

## Results

### Description of socio-demographic characteristics of the respondents by sex

This study enrolled 694 participants with response rate 99.14%. Among them 460 were males; mean age 31 ± 11.17 yrs. (range 18 to75); mean age of females was 32.46 ± 11.17 years (range 18 to 72 year). Most participants (70.0%) were in relationship and 56.6% of respondents have children. Around 38% of participants were employee; more females (33.8%) were employed than males (29.3%). About 38.2% of respondents had secondary level of education. Thirty six (6.5%) respondents had college degree & above, only six; 2.6%; of them were females (Socio-demographic characteristics of the respondents by sex (Table 1)).

**Table 1:** Socio-demographic characteristics of patient with epilepsy by sex at Amanuel Mental Specialized Hospital: Addis Ababa, Ethiopia - 2016

Variable	Over all sample (*n* = 694)	Females (*n* = 234)	Males (*n* = 460)
Sex	694	234 (33.7%)	460 (66.3)
Age			
< 21	69 (9.9)	34 (14.5)	35 (7.6)
21–30	284 (40.9)	91 (38.9)	193 (42.0)
31–40	195 (28.1)	64 (27.4)	131 (28.5)
41–50	89 (12.8)	28 (12)	61 (13.3)
> 50	57 (8.2)	17 (7.3)	40 (8.7)
Ethnicity: n (%)			
Oromo	204 (29.4)	68 (29.1)	136 (29.6)
Amhara	271 (39.0)	89 (38.0%)	182 (39.6)
Guragie	140 (20.2)	50 (21.4)	90 (19.6)
Other	79 (11.4)	27 (11.5)	52 (11.3)
Religion: n (%)			
Orthodox	466 (67.1)	155 (66.2)	311 (67.6)
Muslim	139 (20.0)	42 (17.9)	97 (21.1)
Protestant	82 (11.8)	33 (14.1)	49 (10.7)
Other	7 (1.0)	4 (1.7)	3 (0.6)
Relationship status: n (%)			
In relationship	486 (70.0)	167 (34.4)	319 (65.6)
Not in relationship	208 (30.0)	67 (28.6)	141 (30.7)
Occupation: n (%)			
Employee	214 (30.8)	79 (33.8)	135 (29.3)
Jobless	159 (22.9)	71 (30.3)	88 (19.1)
Self-employee	190 (27.4)	24 (10.3)	116 (36.1)
House wife	43 (6.2)	43 (17.6)	–
Student	32 (4.6)	12 (5.1)	20 (4.3)
Farmer	56 (8.1)	9 (3.8)	47 (10.2)
Education: n (%)			
No formal education	108 (15.6)	48 (20.6)	60 (13.0)
Primary school	218 (31.5)	68 (29.2)	150 (32.6)
Secondary S.	265 (38.2)	90 (38.6)	175 (38.0)
Diploma	66 (9.5)	21 (9.0)	45 (9.8)
Degree & above	36 (5.2)	6 (2.6)	30 (6.5)
Have Children: n (%)			
Yes	393 (56.6)	138 (59.0)	255 (55.4)
No	301 (43.4)	96 (41.0)	205 (44.6)

### Description of psychological factors among patient with epilepsy by sex

From all participants 50 (7.2%) have substance use behavior. Among them eight were female. Khat is more frequently (9.6%) used substance than cigarette (2.1%) and alcohol (3.3%). Almost all (98.6%) participants have good self esteem. Majority (83.1%) of respondents were experienced stressful life events in the past six months. Female participants were slightly stressful than males (88.5% compared to 80%). Most (41.2%) of participants are satisfied with non-sexual aspects of their relationship (Psychosocial characteristics of participants –Table 2).

**Table 2:** Description of psychosocial characteristics of patient with epilepsy at Amanuel Mental Specialized Hospital: Addis Ababa, Ethiopia; 2016

Variables	Over all sample:*n* = 694	Females:*n* = 234	Males: *n* = 460	
Substance use: n (%)				
Yes	50 (7.20)	8 (3.40)	42 (9.10)	
No	644 (92.80)	226 (96.60)	418 (90.90)	
Types of substance: n (%)				
Khat	38 (9.60)	6(2.60)	32 (7.00)	
Cigarette	12 (2.10)	3 (2.20)	9 (1.80)	
Alcohol	19 (3.30)	2 (1.20)	17 (4.20)	
Stressful life events: n (%)				
Yes	577 (83.10)	207 (88.50)	370 (80.40)	
No	117 (16.90)	27 (11.50)	90 (19.60)	
Self-esteem: n (%)				
Good	568 (98.60)	167 (97.7)	401 (99.00)	
Poor	8 (1.40)	4 (2.30)	4 (1.00)	
Relationship satisfaction: n (%);				
Satisfied	286 (41.20)	90 (38.50)	196 (42.60)	
Not satisfied	200 (28.80)	77 (32.90)	123 (26.70)	
Not in relationship	208 (30.00)	67 (28.6)	141 (30.70)	

### Description of clinical factors among patient with epilepsy by sex

Epilepsy began between 6 months and 57 years of age (mean 18.28 ± 11.57). Among all participants 38.9% experienced seizure attacks in the past one month of the study period. Female participants were experiencing seizure attacks more than males (43.2% compare to 36.7%). Twenty six percent of participants have controlled seizure. Among them 70% are males. In medication regimen; 76.1% PWEs were under monotherapy. Phenobarbitone was the most commonly prescribed and combined AED. Twenty four percent of participants have co-morbid illness. Hypertension was more frequent (6.2%) medical illness followed by diabetics (4.9%) and HIV/AIDS (5.9%). Depression was more frequent mental illness (4.9%) than psychosis (3.3%), and it has equal distribution among female and male participants (7.3%). Majority of participants (74.2%) were in normal ranges of BMI, female participants were more underweight than males (13.7% compare to 8.9%) (Clinical factors of participants – Table 3).

**Table 3:** Distribution of clinical factors by sex among patient with epilepsy at Amanuel Mental Specialized Hospital: Addis Ababa, Ethiopia; 2016

Variable	Over all sample: *n*=694	Female: *n*=234	Male: *n*=460
Age onset of Epilepsy: n (%)			
< 5years	97 (14.00)	33 (14.10)	64 (13.90)
5 – 14	167 (24.10)	62 (26.50)	105 (22.80)
15 – 24	253 (36.50)	74 (31.60)	179 (38.90)
25 – 34	99 (14.30)	42 (17.90)	57 (12.40)
35 – 44	62 (8.90)	20 (8.50)	42 (9.10)
>44	16 (2.30)	3 (1.30)	13 (9.10)
Seizure experience			
Not controlled			
Severe	270 (38.90)	101(43.20)	169 (36.70)
Moderate	72 (10.40)	27 (11.50)	45 (9.80)
Mild	169 (24.40)	52 (22.20)	117 (25.40)
Controlled	183 (26.40)	54 (23.10)	129 (28.00)
Medication Regimens: n (%)			
Mono therapy	541 (78.10)	178 (76.10)	363 (79.10)
Poly therapy	152 (21.90)	56 (23.90)	96 (20.90)
Types of medication: n (%)			
Phenobarbitone	557 (80.30)	197 (84.20)	360 (78.30)
Carbamazepine	128 (18.40)	34 (14.50)	94 (20.40)
Phenytoin	94 (13.50)	33 (41.10)	61 (13.30)
Na-valporate	53 (7.60)	20 (8.50)	33 (7.20)
Co morbid illness: n (%)			
Yes	170 (24.50)	59 (25.20)	111 (24.10)
No	524 (75.50)	175 (74.80)	349 (75.90)
Medical co-morbidities: n (%)			
Hypertension	43 (6.20)	22 (9.40)	21 (4.60)
Diabetics	36 (5.20)	21 (9.00)	15 (3.30)
HIV	41(5.90)	30 (12.80)	11 (2.40)
Other	9 (1.30)	5 (2.10)	4 (0.90)
Psychiatric co-morbidities: n (%)			
Psychosis	21 (3.00)	9 (3.80)	12 (2.60)
Depression	34 (4.90)	17 (7.30)	17 (7.30)
Medication for co-morbid illness: n (%)			
Yes	82 (11.80)	41 (17.50)	41 (8.90)
No	611 (88.00)	193 (82.50)	419 (91.00%)
BMI: n (%)			
Under weight	73 (10.50)	32 (13.70)	41 (8.90)
Normal weight	515 (74.20)	156 (66.70)	359 (78.00)
Over weight	106 (15.30)	46 (19.70)	60 (13.00)

### Prevalence of sexual dysfunction among patient with epilepsy

Among all (694) respondents, 583 (84%) had sexual experience for six months and above from data collection date. Among those 583, 576 (98.79%) were completed the CSFQ-14 all items & they were included in the analysis part of global sexual dysfunction, sexual pain problem (dyspareunia), sexual pleasure and orgasmic dysfunction. And other domains of sexual dysfunction; sexual desire and sexual arousal problem; were analyzed based on 694 (99.14%) respondents. More males (70.26%) than females (29.74%) provided data for Changes in Sexual Functioning Questionnaire (CSFQ-14).

### Prevalence of global sexual dysfunction among PWE

**Fig. 1:**
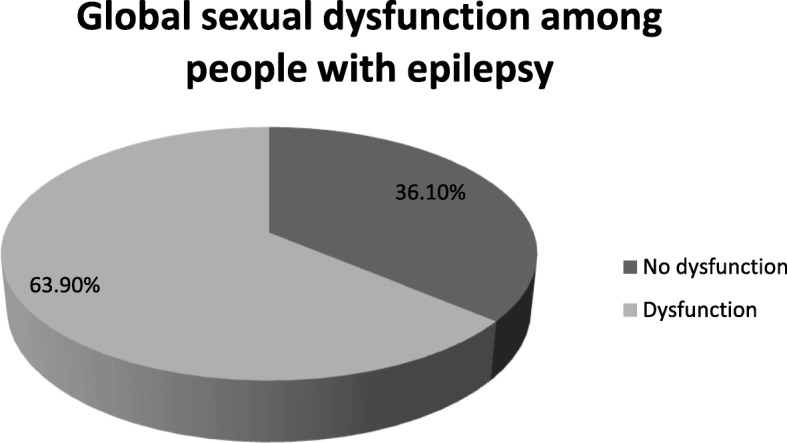
Prevalence of global sexual dysfynction among people with epilepsy at Amanuel Mental Specialized hospital: Addis Ababa; Ethiopia- 2016 (*n* = 576)

Prevalence of global sexual dysfunction among patient with epilepsy is 63.9% (95% CI = 59.5–67.7). It is more prevalent among males than that of females (67.4% (95%CI = 62.8–72.1) to 55.6% (95% CI = 49.1–62.6)) (Fig. 1).

### Prevalence of sexual dysfunction in each domains of sexual dysfunction

**Fig. 2:**
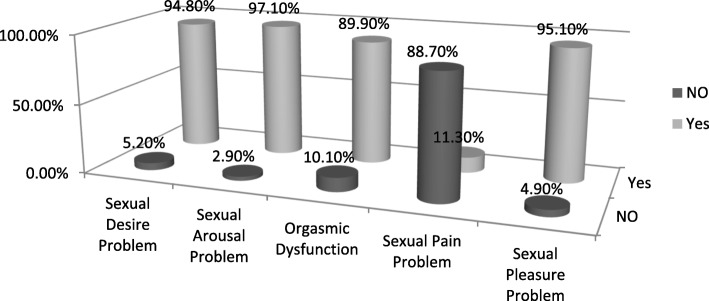
Prevalence of sexual dysfunction in each domains of Sexual Dysfunction among PWE at AMSH, A.A.-Ethiopia; 2016

In each domain; prevalence of sexual arousal problem among PWE is higher; 97.8, 95%CI; 95.8–98.3; and sexual pain problem is lower; 11.3, 95%CI; 8.8–13.9; than other domains (Fig. 2).

### Prevalence of domains of sexual dysfunction by sex

**Fig. 3:**
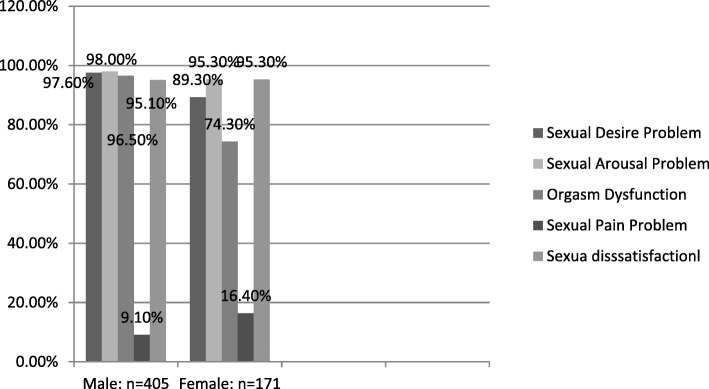
Prevalence of sexual dysfunction in each domains by sex at AMSH, A.A.-Ethiopia; 2016

Sexual pain problem is the least prevalent; 6.4% (95% CI; 11.7–23.2) for females & 9.1% (95% CI; 6.2–11.9) for males; domain of sexual dysfunction in both sex categories. Other domains are highly prevalent among both sex categories (Fig. 3).

### Factors associated with sexual dysfunction among patient with epilepsy

The binary logistic (crude analysis) was done on socio-demographic variables (Age, relationship status, occupation, educational status), clinical variables (depression, psychosis, AED; Phenytoin, carbamazepine, Na valporate and phenobarbitone; medication regimen, body mass, episode of seizure, age onset of epilepsy) and psychosocial variables (current alcohol use, khat use, cigarette use, stressful life event/s, relationship satisfaction and self-esteem).

Variables that found to be *p*-value of less than 0.2 in Univariate logistic regression (age, level of education, occupation, stressful life event, relationship status, khat adulis use, alcohol use, cigarette use, type of medication and medication regimen, age onset of epilepsy, seizure experience, and obesity) are taken in to multivariable logistic regression. The variable group expected to be protective against sexual dysfunction is treated as the reference group.

The odds of developing sexual dysfunction decreased by 74%; (AOR = 0.26, 95% CI = 0.12–0.60); among participants in the age group under 21. But it increased 3.23 times; (AOR = 3.23, 95%CI; 1.21–8.67); among the age group above 51.

The odds of developing sexual dysfunction is decreased by 65%; (AOR = 0.35, 95%CI = 0.17–0.71); and 73%; (AOR = 0.30, 95%CI = 0.12–0.72); among level of education at 2^ndry^ and diploma levels when it compared with those at 1^ry^ level.

The odds of developing sexual dysfunction were decreased by 74%; (AOR = 0.26; 95% CI = 0.075–0.87); in alcohol users compared with not alcohol users.

In the variable occupation, the odds of developing sexual dysfunction is increased 2.59; (AOR = 2.59, 95% CI = 1.34–5.03); times among jobless when it compared with the employed one.

The odds of developing sexual dysfunction is 6.28; (AOR = 6.28, 95%CI = 1.67–23.61); times increased among depressed patients when it compared with people without depression.

The risk of developing sexual dysfunction is 4.10; (AOR = 4.1, 95%CI = 1.43–11.77); times increased among khat users when it compared with non users.

The risk of developing sexual dysfunction is increased 2.62; (AOR = 2.62, 95%CI = 1.62–4.23); and 4.76; (AOR = 4.76, 95%CI = 2.50–9.04); times among those who were not satisfied with their relationship status and among those who are not in relationship when compared with those who were satisfied with their relationship status.

However, in this study there is no significant association identified between the duration of illness, age onset of epilepsy, type of medication, medication regimen, seizure experience, comorbid medical illness, sex, marital status, BMI, life threatening events, self-steam and cigarette use (Table 4).

**Table 4:** Factors which has association on binary logistic regression among patient with epilepsy: Amanuel Mental Specialized Hospital, Addis Ababa, Ethiopia- 2016

Variables	Results: *n*=576			
	Sexual dysfunction		COR (CI=95%)	AOR (CI=95%)
	Yes	No		
Socio-demographic factors				
Age				
<21	20	20	0.52 (0.26 – 1.01)*	0.26 (.012-0.60)**
21 – 30	159	82	1.00	1.00
31 – 40	112	56	1.03 (0.68 – 1.56)	1.44 (0.85-2.42)
41 – 50	34	40	0.44 (0.26 – 0.74)**	0.68 (0.33 – 1.39)
>51	43	10	2.22 (1.06 – 4.64)*	3.23 (1.21 - 8.66)*
Marital status				
Married	171	77	1.00	1.00
Single	177	121	0.66 (0.46 - 0.94)*	0.66 (0.40 – 1.08)
Divorced	7	5	0.63 (0.19 – 2.05)	0.29 (0.62-1.34)
Other	13	5	1.17(0.40 - 3.40)	0.45(0.12 – 1.65)
Educational Status				
Primary	65	24	1.00	1.00
No formal education	118	60	0.73 (0.41 – 1.30)	0.52(0.26-1.03)
Secondary	136	84	0.60 (0.35 – 1.02)*	0.35(0.17-0.71) **
Diploma	31	27	0.42 (0.21 -0.85)**	0.29(0.12-0.72) **
>=Degree	17	13	0.48 (0.20- 1.19)*	0.42 (0.15-1.19)
Occupation				
Employee	108	83	1.00	1.00
Jobless	80	24	2.65 (1.50 – 4.39)**	2.64 (1.36-5.12) **
Self employee	123	50	1.89 (1.22 – 2.92)*	1.70(1.02 -2.83)*
House wife	20	19	0.81 (0.41- 1.61)	0.96(0.42-6.02)
Student	11	7	1.21 (0.45 -3.25)	1.64(0.45-6.03)
Farmer	26	25	0.80 (0.43 – 1.48)	0.70(0.32-1.52)
Clinical factors				
Depression				
Yes	23	5	2.71(1.01 – 7.23)*	6.28(1.67-23.61)**
No	345	203	1.00	1.00
Anti Epileptic Drugs				
Phenobarbitone				
Yes	294	167	0.91 (0.64 – 1.50)	-
No	74	41	1.00	-
Carbamazepine				
Yes	70	32	0.27 (0.82 – 2.04)	0.31 (0.80 – 2.01)
No	298	176	1.00	1.0.
Phenytoin				
Yes	41	33	0.11 (0.41 – 1.09)*	0.12 (0.41 – 1.11)
No	327	175	1.00	1.00
Na – valporate				
Yes	24	18	0.35 (0.39 – 1.39)	-
No	18	190	1.00	-
Current Substance use behaviors				
Khat				
Yes	26	7	2.18 (0.93 – 5.12)*	4.10(1.43-11.77)**
No	342		1.00	1.00
Alcohol				
Yes	9	10	0.5 (0.2-1.24)*	0.26(0.08- 0.87)*
No	359		1.00	1.00
Stressful life events				
Yes	293	183	0.53 (0.33- 0.87)*	0.59(0.32-1.04)
No	75	25	1.00	1.00
Relationship status (couple’s satisfaction)				
Satisfied	146	137	1.00	1.00
Not satisfied	130	49	2.49 (1.66 – 3.73)***	2.62 (1.632 – 4.23)***
Not in relationship	92	22	3.92(2.33 – 6.60)***	4.76 (2.56 – 9.04)***

N.B. * = *p*-value< 0.05, **p-value< 0.01, ***p-value< 0.001

*COR*  Crude Odds Ratio, *AOR*   Adjusted Odds Ratio

## Discussion

In this study; the prevalence and possible factors associated with sexual dysfunction among PWE were assessed; as the result indicated the prevalence of global sexual dysfunction found higher and it is more prevalent among males than females.

This finding is higher when compared with the study conducted in Los Angeles and Egypt (50 and 59.3% respectively) [10, 15]. The possible reason might be differences in; study design (those studies use case and control), sample size (60 cases in Los Angeles and 27cases in Egypt), and variance in socioeconomic characteristics of participants.

The result of sexual dysfunction among male participants was relatively higher (67%) when it compared with the study conduct in Egypt (61%). The possible reasons may the Egypt one was experimental study (investigating Serological and psychological interviews together with serum total testosterone) [16].

Among domains of global sexual dysfunctions, arousal dysfunction has slightly higher prevalent than other domains of sexual dysfunctions; pleasure dysfunction, sexual desire problem and orgasm dysfunction. By the other side dyspareunia was less prevalent sexual dysfunction.

The result of arousal dysfunction among male participants was very high in this study when it compared with other studies conducted on patient with epilepsy. In Iran the prevalence of erectile dysfunction (ED) was 42.5% which is less compared with the results from Massachusetts 52% [17, 18]. In Egypt; erectile dysfunction (ED) and premature ejaculation represented 70.4 and 66.7%, respectively [16]. This difference may be due to cultural difference, availabilities of clinical services and variance in attitude and practices of professionals & participants among study populations.

The study conducted in India; Sri Shivarathreeshwara Hospital was disclosed that; prevalence of clinically significant impairment in different domains of sexual dysfunction is 2–3 times higher in women with epilepsy. This prevalence ranges from 70% (impaired desire) to 98% (impairments in orgasmic functioning) in individual sexual domains, and is 72% for the prevalence of clinically significant impaired global sexual functioning. In this study; its prevalence is higher in desire part and lower in orgasmic dysfunction among females. In global prevalence of clinically significant impaired sexual functioning among females, its prevalence is lower in this study. These differences might happen because of socio-cultural contexts of study participants in this study to disclose ones sexual experiences [19].

The study conducted in North America report recurrent pain during intercourse is in line (15%) with prevalence of dyspareunia among FWE in this study [20]. High prevalence of sexual pain problem is not common among MWE as well in general population. In this study unusual increased in prevalence of sexual pain problem among MWE might be probably because of assessment toll (a single question with above average cut of point; 4 and less out of 5) to screen sexual pain problem.

The odds of developing sexual dysfunction is increased 3.23 times among age groups above 51 years old than in the age groups 21–30.

The odds of developing sexual dysfunction are 6.3 times increased among depressed patients when it compared with people without depression.

The odds of developing sexual dysfunction are 2.64 times increased among jobless when it compared with the employed one. In the variable participants educational status, the odds of developing sexual dysfunction is decreased by 66% among educational level at 2^ndry^ School compared with those at primary level. Also the odds of developing sexual dysfunction were decreased by 70% among diploma holders. It may because of having better awareness about communication, their disease control, sexual life and sexuality among more educated groups than groups at primary level of education.

In this study the risk of developing sexual dysfunction is increased 2.61 times among participants who were not satisfied with non-sexual aspects of their relationship than those who were satisfied. Also its odds were increased 4.76 times among who is not in relationship despite they have sexual experience. This association may occur because of instability on relationship, felling of insecurity, and having sexual practice with strangers. Also impaired sexual dysfunction probably may predispose PWE to be not in relationship. This causal association cannot be made on cross sectional data. Participants those satisfied in non-sexual aspects of relationship may relatively free from psychological factors relating intimacy/relationship and sexuality. According to synopsis of psychiatry 11th edition; the availability of an appropriate partner and a good relationship in non-sexual areas with a partner are among factors affecting once sexual functioning. Damage to, or absence of, any of these factors can diminish desire [16].

Other studies were shown that most of the psychological elements; Self-esteem, quality of relationship, stressful life events; that can interfere in a sexual response can behave in an indiscriminate manner in individuals with or without epilepsy, but in the case of PWE, it appears that the negative-subjective attribution related to “being sick” produces certain behavioral problems that interfere with the sexual response [1, 2, 10, 21].

The odds of developing sexual dysfunction among alcohol users are decreased by 74% when it compared with non users. In this study amount, frequency, concentration and alcohol use behavior of participants were not assessed in detailed and this protective effects of alcohol might occur because of a relaxant effect of it in small amounts. As synopsis of psychiatry 11th edition discussed;

*Abused recreational substances affect sexual function in various ways. In small doses, many substances enhance sexual performance by decreasing inhibition or anxiety or by causing a temporary elevation of mood. Alcohol may foster the initiation of sexual activity by removing inhibition, but it also impairs performance. With continued use, however, erectile engorgement and orgasmic and ejaculatory capacities become impaired* [5, 16]*.*

In this case detailed study is important in alcohol use behaviors of study participants to clarify its effect. Most studies find that, PWE are prone to anxiety and inhibition due to their illnes than people without epilepsy [2, 5, 10]. That problem may lead them to use small amount of alcohol to alleviate their anxiety and inhibition at the time of sexual intercourse. This may be another reason behind this negative association between sexual dysfunction and alcohol use.

The risk of developing sexual dysfunction is 4.1 times increased among khat users when it compared with non users. Meta analysis conducted on published data and limited interviews of regular khat users at East African’s revealed that khat chewing lowers libido in humans and may also lead to sexual impotence following long term use [16].

Type of AEDs (Phenobarbitone, Phenytoin, Carbamazepine, and valporate), medication regimen (polytherapy), age onset of epilepsy, and frequency of seizure experience, body mass, self-esteem, and other variables do not appear to have an independent effect on global sexual functioning.

## Conclusion

The prevalence of global sexual dysfunction among patient with epilepsy found to be higher at Amanuel Mental Specialized Hospital PWE Outpatient clinics. Its prevalence is more on male with epilepsy than females. Among domains of sexual dysfunctions arousal and sexual pain problems were the most & the least prevalent types of sexual dysfunctions respectively. This shows that; it is significant clinical health problem that require a great emphasis.

Although further research will be needed to elucidate the underlying mechanisms of association, this study weighted the data from AMSH, 2016 to succeed in identifying a statistically significant association between sexual dysfunction and the following variables; age, joblessness, level of education, depression, relationship satisfaction, khat use and alcohol use. AEDs and medication regimen (polytherapy) was among variables found as statistically significant in the binary logistic (crude analysis) but it was not statistically significant in multivariable logistic regression.

## Additional files

Additional file 1: Dataset for each domains of sexual dysfunction. (CSV 154 kb)

Additional file 2: Dataset for global sexual dysfunction among patient with epilepsy. (CSV 125 kb)

Additional file 3: Dataset for sexual dysfunction among female participants. (CSV 54 kb)

Additional file 4: Dataset for sexual dysfunction among male participants. (CSV 106 kb)

## Data Availability

The datasets generated and analyzed during this study are available as Additional files 1, 2, 3, 4.

## Abbreviations

AA: Addis Ababa
AEDs: Anti Epileptic Drugs
AMSH: Amanuel Mental Specialized Hospital
BMI: Body Mass Index
CSI: Couple Satisfaction Index
DSLEM: Daily Stressful Life Events Measurement
ED: Erectile Dysfunction
Epi.Info: Epidemiological Information
FEW: Female with Epilepsy
HIV/AIDS: Human Immune compromising viruses/ Acquired Immune Deficiency Disorders
Kg: Kilo gram
m^2^: Meter square
MWE: Male with Epilepsy
NEP: Neuropsychiatric
NHSLS: National Health and Social Survey
OPD: Out Patient Department
PE: Premature Ejaculation
PWE: Patient with Epilepsy
PWE: Peoples with Epilepsy
RAS: Relationship Assessment Scale
SD: Sexual dysfunction
SES: Self-esteem Scale SLE: Stressful life events
SPSS: Statistical Package for Social Science
TLE: Temporal Lobe Epilepsy
UOG: University of Gondar
